# SAA1 regulated by S1P/S1PR1 promotes the progression of ESCC via β-catenin activation

**DOI:** 10.1007/s12672-024-00923-3

**Published:** 2024-03-06

**Authors:** Qianqian Li, Maolin Tang, Shisheng Zhao, Junjie Yang, Yuanlin Meng, Chunmei Meng, Ling Ren, Weimin Hu

**Affiliations:** 1 Institute of Basic Medicine and Forensic Medicine, North Sichuan Medical College, Nanchong, 637100 China; 2 Department of Immunology, North Sichuan Medical College, Nanchong, 637100 China

**Keywords:** Serum amyloid A1 (SAA1), Esophageal squamous cell carcinoma (ESCC), pSer675-β-catenin, S1P/S1PR1

## Abstract

Serum amyloid A1 (SAA1), an inflammation-related molecule, is associated with the malignant progression of many tumors. This study aimed to investigate the role of SAA1 in the progression of esophageal squamous cell carcinoma (ESCC) and its molecular mechanisms. The expression of SAA1 in ESCC tissues and cell lines was analyzed using bioinformatics analysis, western blotting, and reverse transcription-quantitative PCR (RT‒qPCR). SAA1-overexpressing or SAA1-knockdown ESCC cells were used to assess the effects of SAA1 on the proliferation, migration, apoptosis of cancer cells and the growth of xenograft tumors in nude mice. Western blotting, immunofluorescence and RT‒qPCR were used to investigate the relationship between SAA1 and β-catenin and SAA1 and sphingosine 1-phosphate (S1P)/sphingosine 1-phosphate receptor 1 (S1PR1). SAA1 was highly expressed in ESCC tissues and cell lines. Overexpression of SAA1 significantly promoted the proliferation, migration and the growth of tumors in nude mice. Knockdown of SAA1 had the opposite effects and promoted the apoptosis of ESCC cells. Moreover, SAA1 overexpression promoted the phosphorylation of β-catenin at Ser675 and increased the expression levels of the β-catenin target genes MYC and MMP9. Knockdown of SAA1 had the opposite effects. S1P/S1PR1 upregulated SAA1 expression and β-catenin phosphorylation at Ser675 in ESCC cells. In conclusion, SAA1 promotes the progression of ESCC by increasing β-catenin phosphorylation at Ser675, and the S1P/S1PR1 pathway plays an important role in its upstream regulation.

## Introduction

Esophageal cancer (EC) is a major global health challenge. The latest global statistics show that EC is the sixth leading cause of cancer-related death and the seventh most common cancer worldwide [1]. Although great progress has been made in the treatment of EC in recent years, the prognosis is still poor. In 2010–2014, the 5 year age-standardized survival rate was in the range of 10–30% in most countries [2]. Esophageal squamous cell carcinoma (ESCC) is the main pathological type of esophageal cancer, especially in East Asia and eastern and southern Africa [3, 4]. Inflammation plays an important role in the carcinogenesis of ESCC by cultivating a microenvironment conducive to tumorigenesis [5].

Serum amyloid A1 (SAA1), a member of the SAA family (SAA1, SAA2, SAA3 and SAA4), is significantly upregulated during acute inflammation and tissue damage in the body [6, 7]. SAA1 is involved in the pathogenesis of many inflammatory diseases [8, 9]. A growing body of data supports the notion that SAA1 is involved in carcinogenesis [10]. Several studies have shown that SAA1, as a nonspecific tumor marker and independent prognostic factor, plays an important role in the diagnosis and prognosis of ovarian cancer, lung cancer, colorectal cancer, pancreatic cancer, renal cancer and other tumors [11–14]. SAA1 is highly expressed in ESCC tissue and is associated with poor prognosis [15, 16]. However, few of these studies directly address the mechanisms by which SAA1 promote the progression of cancer.

β-catenin, as an important component of cadherin-based adherens junctions, plays a vital role in maintaining the integrity of the organism [17]. Moreover, β-catenin is a key transcription coactivator in the canonical Wnt cascade, responsible for signal transduction to the nucleus and activation of transcription factors that trigger downstream target gene transcription, such as c-Myc, cyclinD1, MMP-9, Snail, and ZEB1 by binding to T-cell factor (TCF)/lymphoid enhancer-binding factor (LEF) transcription factors [17, 18]. The functional output of β-catenin is affected by posttranslational modifications, and phosphorylation is an important part of this process [19]. It is currently believed that phosphorylation at Ser675 promotes the stabilization and nuclear translocation of β-catenin and positively regulates the Wnt cascade [17]. Sustained activation of β-catenin leads to the continuous self-renewal and growth of tumor cells and is associated with therapeutic resistance [17, 18]. Some studies have suggested that the β-catenin signaling pathway plays a key role in promoting the proliferation of ESCC cells [20]. The regulatory relationship between SAA1 and β-catenin in tumors has not been reported.

Sphingosine 1-phosphate (S1P) is a bioactive lipid produced by the phosphorylation of sphingosine by sphingosine kinase (SPHK). Secreted extracellular S1P transmits signals by binding to five G-protein-coupled receptors (S1PR1, S1PR2, S1PR3, S1PR4 and S1PR5) to regulate cell differentiation, proliferation, migration, and survival [21]. S1P/S1PR1 signaling plays an important role in the progression of various tumors [22, 23]. Pan et al. found that the expression of SPHK1 in ESCC tissues was higher than that in paratumor tissues, and the exogenous expression of SPHK1 promoted the invasion and metastasis of ESCC cells in vitro and in vivo [24]. Our group has already demonstrated that S1PR1 plays an important role in the proliferation and migration of ESCC cells [25]. Moreover, another study showed that S1P/S1PR1 promotes the nuclear translocation of β-catenin by activating the PI3K/Akt pathway and upregulates the expression of target genes, such as MYC, in osteoblast-like cell lines [26]. The S1P/S1PRs pathway may play a role in promoting tumor progression by regulating β-catenin. Additionally, by RNA sequencing, we found that overexpression of S1PR1 in ESCC cells upregulated the expression of SAA1 (unpublished).

The aim of this study was to investigate the role of SAA1 in the progression of ESCC, and we also sought to determine whether S1P/S1PR1 promotes ESCC progression by regulating SAA1 and activating β-catenin.

## Materials and methods

### Microarray and transcriptome dataset analysis

The mRNA expression level of SAA1 was analyzed in 91 ESCC tissue samples and 11 normal esophageal tissue samples from The Cancer Genome Atlas (TCGA) database (http://www.cancer.gov/tcga). As a supplement, this study also analyzed the expression level of SAA1 mRNA in GSE161533 (28 paired normal tissues, paratumor tissues, and tumor tissues) and GSE44021 (73 paired normal tissues and tumor tissues) from the Gene Expression Omnibus (GEO) database (https://www.ncbi.nlm.nih.gov/geo/).

### Patients and tissue samples

Sixteen pairs of fresh tumor and normal tissues from 16 patients with ESCC were obtained from the Affiliated Hospital of North Sichuan Medical College. All tissues were rapidly frozen in liquid nitrogen after excision and then maintained at − 80 °C. This study was approved by the ethics committee of North Sichuan Medical College (NO. NSMC202161).

### Cell culture and treatment

The human ESCC cell lines Eca109 and TE-1 were obtained from the Cell Bank of Type Culture Collection of the Chinese Academy of Science (Shanghai, China). A human normal esophageal epithelial cell line HEEC was obtained from ScienCell (CA, USA). All cells were cultured in RPMI 1640 medium (Gibco, United States) supplemented with 10% (v/v) fetal bovine serum (Every Green, China), 100 U/mL penicillin and 100 μg/mL streptomycin (HyClone, Austria). All cells were maintained in a standard humidified incubator with 5% CO_2_ at 37 °C.

### Cell transfection and lentivirus infection

The siRNAs against SAA1 (SAA1-siRNA), S1PR1 (S1PR1-siRNA1, S1PR1-siRNA2) and the negative control (NC-siRNA) were synthesized by GenePharma (Shanghai, China). siRNA sequences for SAA1, S1PR1 and NC are shown in Table 1. The S1PR1 overexpression plasmid (OE-S1PR1) and control plasmid (OE-NC) were constructed by Genechem (Shanghai, China). Transient transfection of plasmids and siRNAs was performed with Lipofectamine 2000 (Invitrogen, USA) according to the manufacturer’s instructions. SAA1 overexpression (OE-SAA1) lentivirus and negative control (OE-NC) lentivirus were designed and produced by Genechem (Shanghai, China). TE-1 cells were infected with these lentiviruses and selected with puromycin (Solarbio, China).

**Table 1:** siRNA sequences

	Sequences
NC-siRNA	sense: 5ʹ-UUCUCCGAACGUGUCACGUTT-3ʹ
	antisense: 5ʹ-ACGUGACACGUUCGGAGAATT-3ʹ
SAA1-siRNA	sense: 5ʹ-CUCUUUCCCAACAAGAUUATT-3ʹ
	antisense: 5ʹ-UAAUCUUGUUGGGAAAGAGTT-3ʹ
S1PR1-siRNA1	sense: 5ʹ-CUGACCUGGGUGGUGUUCATT-3ʹ
	antisense: 5ʹ-UGAACACCACCGAGGUCAGTT-3ʹ
S1PR1-siRNA2	sense: 5ʹ-CGGUCUCUGACUACGUCAATT-3ʹ
	antisense: 5ʹ-UUGACGUAGUCAGAGACCGAG-3ʹ

### RNA extraction and reverse transcription-quantitative PCR (RT‒qPCR) analysis

Total RNA was extracted using the SV Total RNA Isolation System (Promega, USA) following the standard procedure. The PrimeScript^™^ RT Reagent Kit with gDNA Eraser (Takara, Japan) was used to obtain cDNA. RT‒qPCR analysis of the samples was performed using CFX Connect (Bio-Rad, USA) with a SYBR Premix ExTaq^™^ II (Tli RNaseH Plus) kit (Takara, Japan). HPRT was used as an endogenous control. The primers used are listed in Table 2. The relative expression of mRNA was computed using the 2^−ΔΔCt^ method.

**Table 2:** RT qPCR primer sequences

Genes	Sequences
SAA1	sense: 5ʹ-GGGAACTATGATGCTGCCAA-3ʹ
	antisense: 5ʹ-TGGCCAAAGAATCTCTGGAT-3ʹ
S1PR1	sense: 5ʹ-GCTGCTCAAGACCGTAATTATCG-3ʹ
	antisense: 5ʹ-ACCAGGAAGTACTCCGCTCTGA-3ʹ
HPRT	sense: 5ʹ-TGAGGATTTGGAAAGGGTGT-3ʹ
	antisense: 5ʹ-GAGCACACAGAGGGCTACAA-3ʹ

### Cell counting kit-8 (CCK-8) assay

ESCC cells were seeded in 96-well plates. The next day, 40% confluent cells were transfected with siRNA. The cells were monitored every 24 h. CCK-8 (Beyotime, China) reagent was added to each well (at a 1:10 dilution). Cells were incubated for another 2 h at 37 °C. Then, the absorbance was measured at a wavelength of 450 nm by an iMark™ microplate reader (Bio-Rad, USA). The CCK-8 assay for the lentivirus-infected cells was the same as described above but without the transfection step after cells were seeded. In the recombinant human SAA1 (rhSAA1, PeproTech, USA) stimulation assay, TE-1 cells were stimulated with 0, 0.1, 1, and 10 µg/mL rhSAA1, and cell proliferation was monitored every 24 h.

### Wound healing assay

Eca109 cells were plated in 12-well plates. After 24 h, the monolayer was scratched with a sterile pipette tip to form a “wound”. The debris was removed by washing twice with phosphate buffered saline (PBS), followed by cell transfection. Finally, images were taken at the same locations at 0, 24 and 48 h after scratching. The degree of cell migration at different times was quantified by the area between the two edges of the wound. The wound healing assay for the lentivirus-infected cells was the same as described above but without the transfection step after cells were seeded. In the rhSAA1 stimulation assay, TE-1 cells were treated with 0 or 10 µg/mL of rhSAA1, and cell migration was monitored for 24 h.

### Colony formation assay

The cells were seeded into 6-well plates (800 cells/well) and cultured for 14 days in an incubator. Next, colonies were fixed with 4% paraformaldehyde for 60 min and stained with 0.5% crystal violet solution. The colonies in three random fields were counted, and the average was calculated.

### Apoptosis assay

TE-1 cell apoptosis was determined using an Annexin V-APC/PI apoptosis assay kit (KeyGEN BioTECH, China) after transfection with SAA1 siRNA. Cells were digested with EDTA-free trypsin, subsequently washed with PBS, collected, and finally stained with Annexin V-APC and PI for 10 min in the dark. Cell apoptosis was analyzed using a flow cytometer (3L13C, ACEA Biosciences, USA).

### Western blot analysis

Cells or tissues were lysed with ice-cold RIPA buffer containing a protease inhibitor mixture. The protein concentration was determined using a BCA protein assay kit (Beyotime, China). Total protein was separated by SDS‒PAGE (8 or 15%). The designated proteins were transferred to pre-cut polyvinylidene difluoride (PVDF) membranes (Millipore, USA). Next, the membranes were blocked in 5% bovine serum albumin (BSA)-TBST for 1.5 h at room temperature and incubated with specific primary antibodies at 4 °C overnight. Then, the membranes were incubated with horseradish peroxidase (HRP)-conjugated secondary antibody (Beyotime, China) for 60 min at room temperature and visualized with a ChemiDoc^™^ XRS + system (Bio-Rad, United States). ImageJ software was used to analyze the relative protein levels. Primary antibodies included those against SAA1 (1:1,000, R&D System, USA), MMP-9 (1:1,000, Proteintech, United States), c-Myc (1:1,000, Proteintech, United States), total β-catenin (1:1,000, CST, United States), and pSer675-β-catenin (1:1,000, CST, United States).

### Immunofluorescence assay

Cells on coverslips were washed twice with PBS buffer and fixed in 4% paraformaldehyde for 15 min at room temperature. Next, the cells were permeabilized in 0.2% Triton for 5 min, washed three times with PBS, blocked in 1% BSA/PBS at room temperature for 1 h, and incubated with the following primary antibodies (β-catenin, diluted 1:100; pSer675-β-catenin, diluted 1:100) overnight at 4 °C. Subsequently, the cells were washed 3 times with PBS and incubated with secondary antibody (goat-anti-rabbit, iFluor^™^ 647, HuaBio, China) diluted 1:500 in PBS containing 1% BSA for 1 h at room temperature. After washing with PBS, cell nuclei were stained with 4′,6 diamidino-2-phenylindole (DAPI) (Solarbio, China) for 5 min and then washed 3 times with PBS. Finally, coverslips were viewed under a confocal laser-scanning microscope (FV3000, Olympus, Japan). All immunofluorescence images within each experiment were captured under the same settings.

### In vivo tumor xenograft model

Ten BALB/c nude mice were purchased from Beijing Huafukang Biotechnology Co., LTD (Beijing, China) and randomly divided into two groups. A total of 2 × 10^6^ stable SAA1-overexpressing TE-1 cells or negative control cells were suspended in 0.1 mL sterilized PBS and then subcutaneously implanted into the left armpit of 5-week-old female BALB/c nude mice (5 mice per group). Tumor volume was measured every 3 days. The animals were sacrificed 3 weeks after transplantation, the tumors were removed, and tumor volume and weight were determined. This study was approved by the ethics committee of North Sichuan Medical College (NO. NSMC202181). Animal study experiments were conducted following the laboratory guidelines for animal care.

### Statistical analysis

All data were analyzed using GraphPad Prism 8 software (GraphPad, CA, USA) and expressed as the mean ± standard deviation (SD). Student’s t test was used to evaluate the differences between groups. p values < 0.05 were considered statistically significant. All experiments were repeated independently at least 3 times.

## Results

### Expression of SAA1 in ESCC tissues and cell lines

**Fig. 1:**
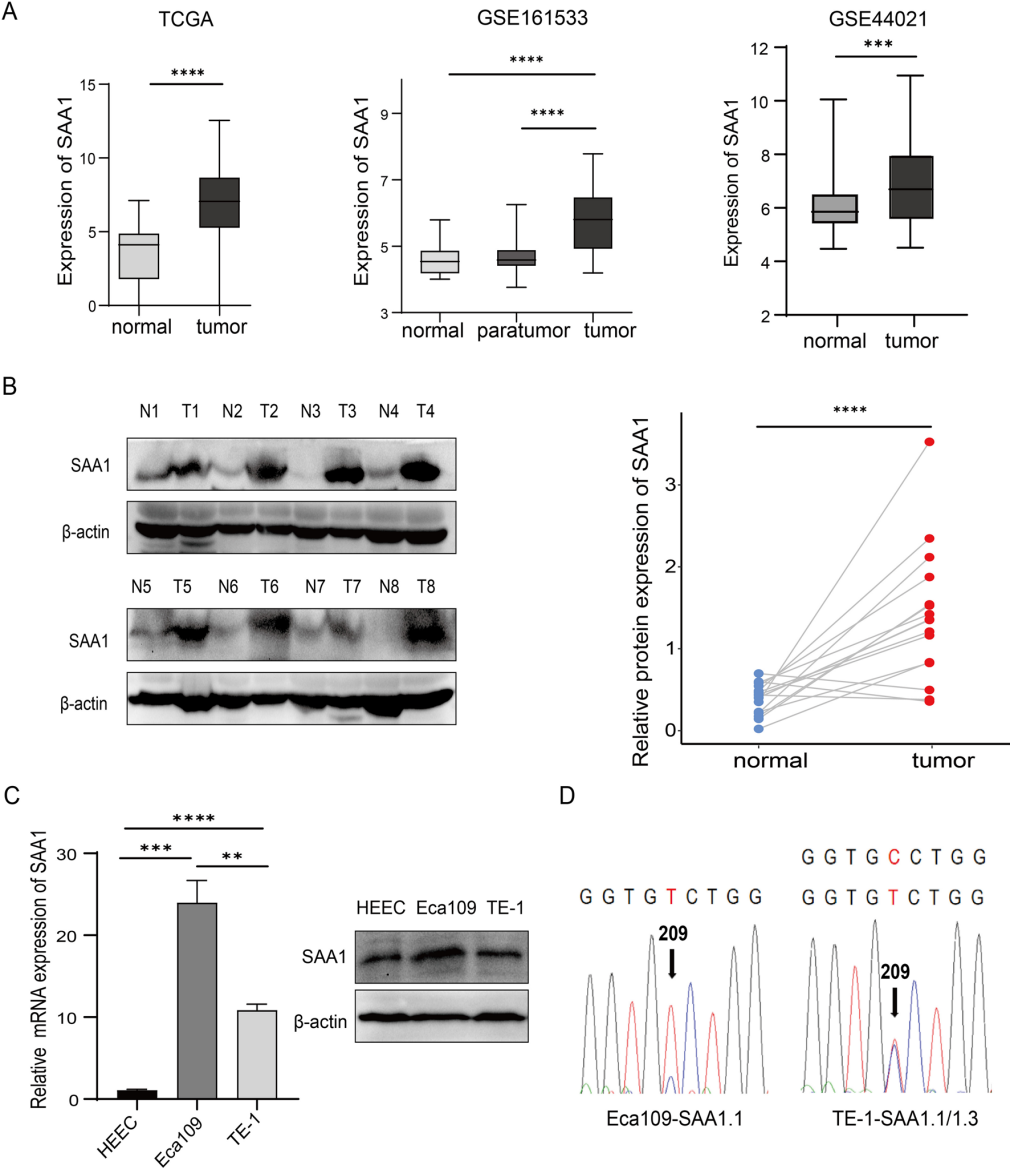
SAA1 is highly expressed in ESCC tissues and cell lines. **A** SAA1 mRNA expression in ESCC tissues in TCGA and GEO datasets was analyzed. **B** SAA1 protein expression in ESCC tissues determined by western blot analysis (n = 16). **C** SAA1 mRNA and protein expression in HEEC cells and ESCC cell lines was determined by RT‒qPCR. **D** The cDNA sequencing results of SAA1 in two ESCC cell lines showed that a single nucleotide polymorphism was observed at nucleotide position 209, as indicated by the arrows. **p < 0.01, ***p < 0.001, ****p < 0.0001

To determine the expression of SAA1 in ESCC tissues, we analyzed an ESCC dataset from TCGA. The SAA1 mRNA level in ESCC tissues was significantly higher than that in normal tissues (Fig. 1A). The raw data of samples in GSE161533 and GSE44021 were downloaded from the GEO database and analyzed. The results showed that SAA1 mRNA levels in ESCC tissues were significantly higher than those in paratumor tissues and normal tissues (Fig. 1A). In fresh tissue samples, SAA1 protein expression was also upregulated in ESCC tissues compared to adjacent normal tissues. Representative results are shown in Fig. 1B.

SAA1 expression in two ESCC cell lines and HEEC cells was evaluated using RT‒qPCR and western blotting. The expression of SAA1 in HEEC cells was lower than that in ESCC cell lines. In addition, the expression of SAA1 was relatively high in Eca109 cells and low in TE-1 cells (Fig. 1C). SAA1 has five polymorphic alleles, SAA1.1, SAA1.2, SAA1.3, SAA1.4 and SAA1.5, encoding distinct proteins with a few amino acid differences [8]. In our study, two SAA1 allelic variants, SAA1.1 and SAA1.3, were identified in ESCC cell lines (Fig. 1D). Both SAA1.1 and SAA1.3 were constitutively expressed in the TE-1 cell line, while only SAA1.1 was expressed in the Eca109 cell line. Moreover, SAA1 polymorphisms have been reported as risk factors in certain inflammatory diseases, and different variants of SAA1 play different roles in cancer [27]. Research shows that SAA1.1 induces stronger ERK phosphorylation than SAA1.3, and ERK phosphorylation is closely related to tumor cell proliferation and migration [28, 29]. SAA1.1 was identified in both TE-1 and Eca109 cell lines, and the commercially available SAA1 is SAA1.1. Therefore, subsequent studies on the overexpression of SAA1 focused on SAA1.1.

### SAA1 promotes the proliferation and migration of TE-1 cells

**Fig. 2:**
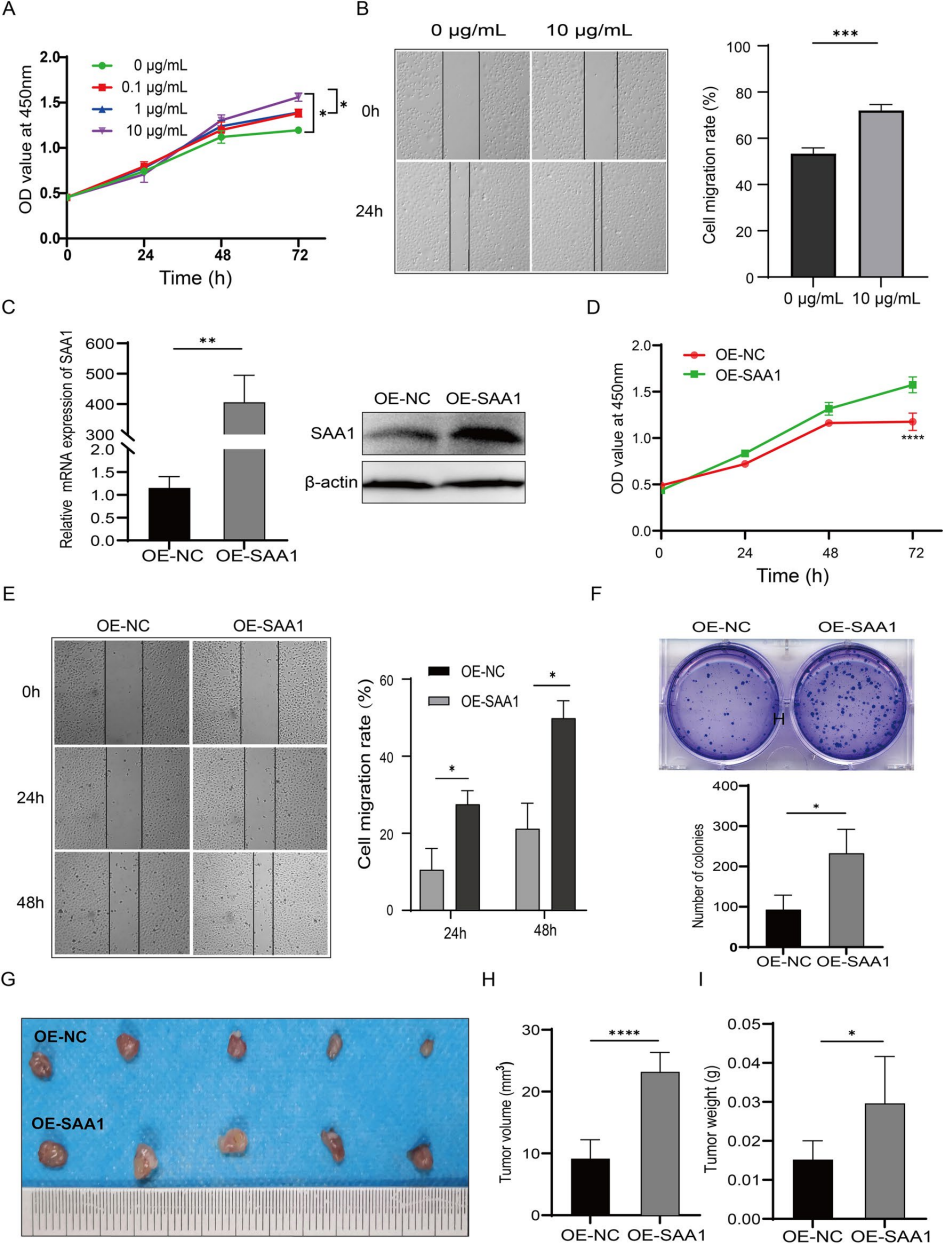
SAA1 promotes the proliferation and migration of TE-1 cells in vitro and in vivo. **A** TE-1 cells were stimulated with different concentrations of rhSAA1 for 72 h, and cell proliferation was determined by CCK-8 assay. **B** TE-1 cells were stimulated with 10 μg/mL rhSAA1 for 24 h, and the migration of cells was determined by wound healing assay. **C** SAA1 was overexpressed in TE-1 cells, and the overexpression efficiencies were confirmed by RT‒qPCR and western blotting. **D** The proliferation of SAA1-overexpressing TE-1 cells was determined by CCK-8 assay. **E** The migration of SAA1-overexpressing TE-1 cells was determined by wound healing assay. **F** The colony formation capacity of SAA1-overexpressing TE-1 cells was determined by colony formation assay. **G** Xenograft tumors were photographed. **H** The volume of xenograft tumors was determined at the end of the experiment. **I** Xenografts were weighed at the end of the experiment. *p < 0.05, **p < 0.01, ***p < 0.001, ****p < 0.0001

According to the expression level results, we selected TE-1 cells for subsequent overexpression functional analysis. To examine the role of SAA1 in the proliferation and migration of ESCC cells, CCK-8 assays and wound healing assays were performed. First, in the rhSAA1 stimulation assay, the results showed that rhSAA1 promoted TE-1 cell proliferation and migration (Fig. 2A, B). Furthermore, SAA1 was overexpressed in TE-1 cells, and the overexpression efficiencies were confirmed by RT‒qPCR and western blot (Fig. 2C). Overexpression of SAA1 promoted the proliferation and migration of TE-1 cells (Fig. 2D, E). The colony formation assay showed that overexpression of SAA1 increased the colony formation ability of TE-1 cells (Fig. 2F).

### SAA1 enhances tumor growth in vivo

We constructed a subcutaneous xenograft tumor model in nude mice to investigate the role of SAA1 in vivo. The results showed that the tumor volume and tumor weight in the SAA1-overexpression group were larger than those in the OE-NC group (Fig. 2G–I). These results indicate that SAA1 significantly promotes the growth of tumors in vivo.

### Knockdown of SAA1 inhibits the proliferation and migration and promotes the apoptosis of ESCC cells in vitro

**Fig. 3:**
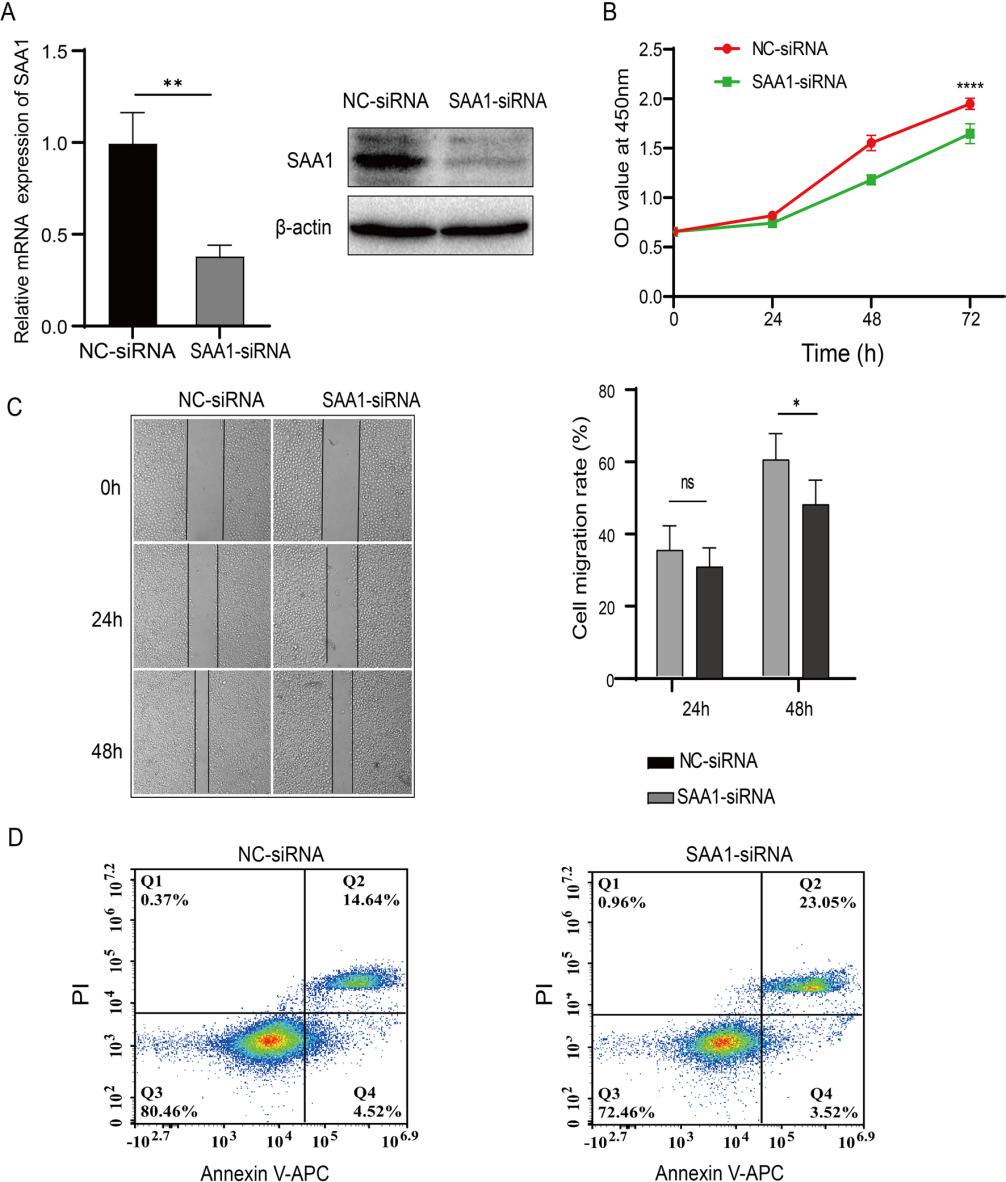
SAA1 knockdown inhibits proliferation and migration, and promotes the apoptosis of ESCC cells. **A** The expression of SAA1 was knocked down in Eca109 cells, and the knockdown efficiencies were confirmed by RT‒qPCR and western blotting. **B** The proliferation of SAA1-knockdown Eca109 cells was determined by CCK-8 assay. **C** The migration of SAA1-knockdown Eca109 cells was determined by wound healing assay. **D** The apoptosis of SAA1-knockdown Eca109 cells was determined by flow cytometry. *p < 0.05, **p < 0.01, ****p < 0.0001

The expression of SAA1 was knocked down in Eca109 cells. Knockdown efficiency was confirmed by RT‒qPCR and western blotting (Fig. 3A). The CCK-8 proliferation assay showed that SAA1 knockdown inhibited the proliferation of Eca109 cells (Fig. 3B). Wound healing assays showed that the migration of Eca109 cells was significantly reduced after SAA1 knockdown (Fig. 3C). The apoptosis assay showed that SAA1 knockdown promoted the apoptosis of Eca109 cells (Fig. 3D). Altogether, these results indicate that SAA1 knockdown inhibits the proliferation and migration and promotes the apoptosis of ESCC cells.

### SAA1 promotes the proliferation and migration of ESCC cells by inducing β‑catenin phosphorylation

**Fig. 4:**
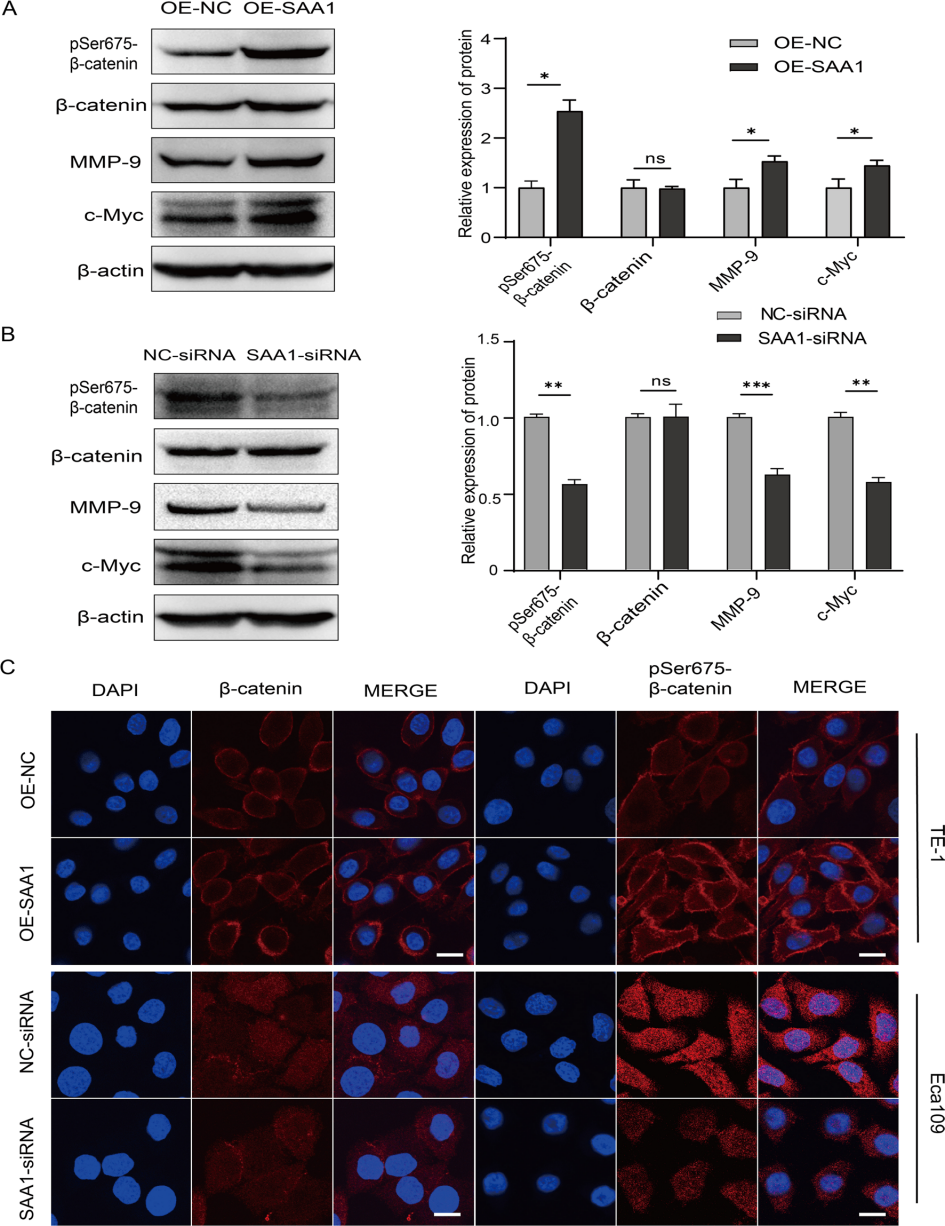
SAA1 induces β-catenin phosphorylation at Ser675. **A** The protein levels of total β-catenin, pSer675-β-catenin, MMP-9 and c-Myc in SAA1-overexpressing TE-1 cells were detected by western blotting. **B** The protein levels of total β-catenin, pSer675-β-catenin, MMP-9 and c-Myc in SAA1-knockdown Eca109 cells were detected by western blotting. **C** TE-1 cells and Eca109 cells were stained with anti-pSer675-β-catenin, anti-β-catenin and DAPI and observed by confocal fluorescence microscopy, scale bar = 20 μm. *p < 0.05, **p < 0.01, ***p < 0.001

**Fig. 5:**
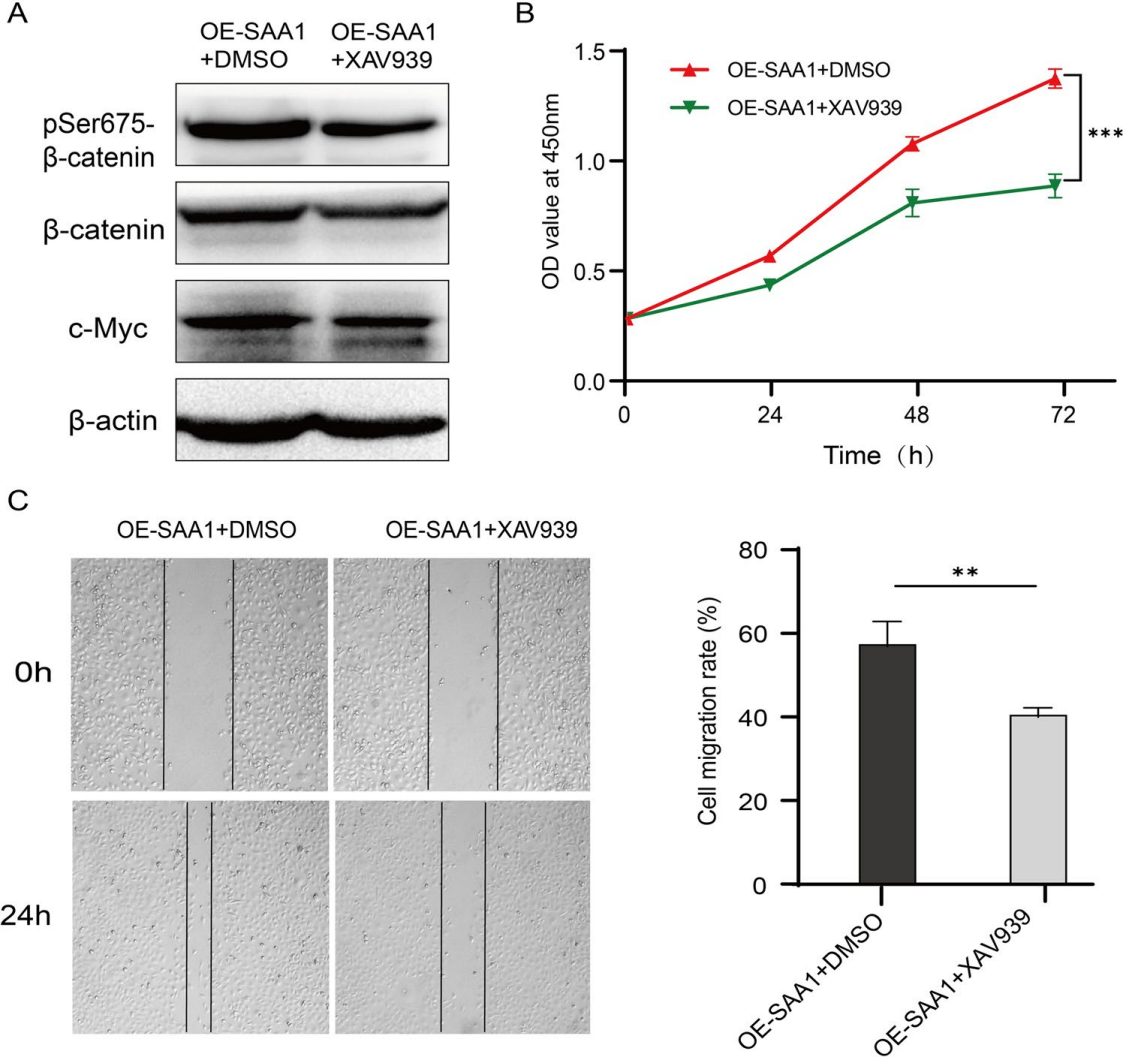
The β-catenin inhibitor XAV939 alleviates SAA1 overexpression-mediated proliferation and migration. **A** SAA1-overexpressing TE-1 cells were treated with XAV939 (10 µmol/L) for 24 h. The protein levels of total β-catenin, pSer675-β-catenin and c-Myc were detected by western blotting. **B** The proliferation of SAA1-overexpressing TE-1 cells treated with XAV939 (10 µmol/L) was determined by CCK-8 assay. **C** The migration of SAA1-overexpressing TE-1 cells treated with XAV939 (10 µmol/L) was determined by wound healing assay. **p < 0.01, ***p < 0.001

To determine whether SAA1 is closely related to β‑catenin, we observed the expression level and sublocalization of β‑catenin in SAA1-overexpressing or SAA1-knockdown ESCC cells. There was no significant change in total β-catenin protein levels after SAA1 overexpression or knockdown (Fig. 4A, B). However, the expression of pSer675-β-catenin was significantly upregulated in SAA1-overexpressing TE-1 cells and significantly downregulated in SAA1-knockdown Eca109 cells. Moreover, SAA1 was positively correlated with the expression of the β-catenin target genes MYC and MMP9 (Fig. 4A, B). In the immunofluorescence assay, β-catenin and pSer675-β-catenin were mainly localized in the cell membrane of TE-1 cells. Small amounts of β-catenin and pSer675-β-catenin were translocated to the cytoplasm and nucleus in SAA1-overexpressing TE-1 cells. There was no significant change in total β-catenin protein levels after SAA1 overexpression in TE-1 cells. However, the expression of pSer675-β-catenin was significantly upregulated in SAA1-overexpressing TE-1 cells (Fig. 4C). β-catenin and pSer675-β-catenin were mainly localized in the cytoplasm and nucleus in Eca109 cells. The nuclear translocation of β-catenin and pSer675-β-catenin was significantly reduced in SAA1-knockdown Eca109 cells. There was no significant change in total β-catenin protein levels after SAA1 knockdown in Eca109 cells. However, the expression of pSer675-β-catenin was significantly downregulated in SAA1-knockdown Eca109 cells. Moreover, after treating SAA1-overexpressing cells with the β‑catenin inhibitor XAV939 (MCE, USA), we found that XAV939 significantly inhibited SAA1-induced Ser675-β-catenin and c-Myc expression (Fig. 5A). Similarly, XAV939 alleviated the proliferation and migration increase mediated by SAA1 overexpression (Fig. 5B, C). Taken together, the effect of SAA1 on promoting the proliferation and migration of ESCC cells is related to β-catenin, which is at least partially mediated by pSer675-β-catenin.

### S1P/S1PR1 increases SAA1 expression levels and β-catenin phosphorylation at Ser675 in ESCC cells

**Fig. 6:**
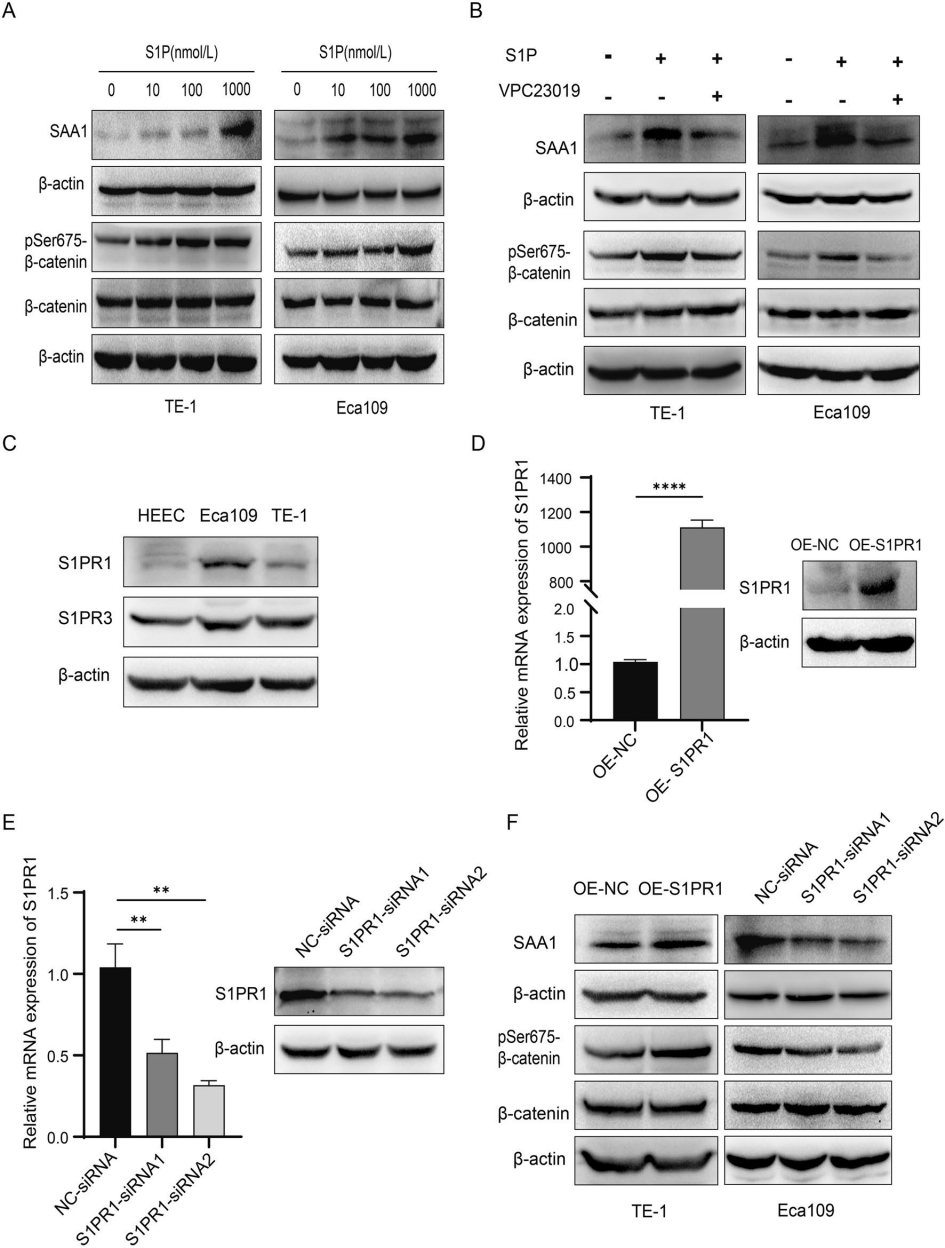
S1P/S1PR1 increases SAA1 expression levels and β-catenin phosphorylation at Ser675 in ESCC cells. **A** ESCC cells were stimulated with different concentrations of S1P (0, 10, 100, and 1000 nmol/L) for 24 h. SAA1, total β-catenin and pSer675-β-catenin protein levels were detected by western blotting. **B** ESCC cells were treated with VPC23019 (1000 nmol/L) and then stimulated with S1P (1000 nmol/L) for 24 h. The levels of SAA1, total β-catenin and pSer675-β-catenin were detected by western blotting. **C** The expression of S1PR1 and S1PR3 proteins in HEEC cells and two ESCC cell lines was detected by western blotting. **D** The efficiency of S1PR1 overexpression was determined by RT‒qPCR and western blotting. **E** The efficiency of S1PR1 knockdown was determined by RT‒qPCR and western blot. **F** The protein levels of SAA1, total β-catenin and pSer675-β-catenin were detected by western blot after S1PR1 overexpression and knockdown in ESCC cells. **p < 0.01, ****p < 0.0001

We determined the expression of SAA1 at different S1P (Cayman Chemical, USA) stimulation concentrations. The protein levels of SAA1 increased in a concentration-dependent manner (Fig. 6A). S1P has five high affinity receptors, and it is generally believed that S1PR1 and S1PR3 promote tumor cell migration and survival. To clarify whether S1P plays a regulatory role in SAA1 by combining with S1PR1 or S1PR3, follow-up experiments were performed. After 12 h of starvation, the cells were treated with VPC23019 (S1PR1/S1PR3 antagonist, Sigma, USA) for 30 min, followed by stimulation with S1P. Pretreatment of TE-1 cells and Eca109 cells with VPC23019 significantly inhibited S1P-induced SAA1 expression. VPC23019 also inhibited the phosphorylation of β-catenin at Ser675 in S1P-treated cells, but the total β-catenin protein expression level was not significantly changed (Fig. 6B).

The expression levels of S1PR1 and S1PR3 in two ESCC cell lines were detected by western blotting. The expression trend of S1PR1 in the two ESCC cell lines was consistent with SAA1, while there was no significant difference in the expression of S1PR3 in the two cell lines (Fig. 6C). Moreover, the results of RNA sequencing suggested that the overexpression of S1PR1 upregulated the expression of SAA1. We hypothesized that S1PR1 plays a more important role than S1PR3 in the regulation of SAA1 expression. Therefore, the S1PR1 overexpression plasmid was used to upregulate the expression of S1PR1 in TE-1 cells, and siRNA was used to knock down the expression of S1PR1 in Eca109 cells. Overexpression or knockdown efficiency was confirmed by RT‒qPCR and western blotting (Fig. 6D, E). The expression of SAA1 and pSer675-β-catenin in the S1PR1-overexpression group was significantly upregulated compared with that in the negative control group in TE-1 cells, while there was no significant change in the total β-catenin expression, and S1PR1 knockdown had the opposite effect (Fig. 6F). These results suggested that S1P/S1PR1 increased the phosphorylation level of β-catenin at the Ser675 site by upregulating the expression of SAA1.

## Discussion

SAA1 is largely regarded as a protumorigenic factor and contributes to tumor initiation and progression via the MAPK/ERK, Akt, NF-κB and IL-1 pathways [13, 30]. Previous studies have shown that SAA1 is involved in the apoptosis, migration, invasion and metastasis of a variety of cancer cells. Lin et al. found that SAA1 promotes glioblastoma multiforme (GBM) cell migration and invasion by combining with integrin αVβ3 and activating the ERK signaling pathway [31]. Another study on GBM showed that SAA1 downregulation can regulate the expression of apoptosis-related proteins by inhibiting AKT phosphorylation, leading to the death of GBM cells [32]. Similarly, Hansen et al. found that S100A4 induces tumor metastasis by targeting SAA1 through the Toll-like receptor 4 (TLR4)/NF-κB signaling pathway [13]. Several studies suggest that SAA may be involved in the progression of ESCC. Wang et al. showed that a high level of preoperative plasma SAA is associated with tumor progression and poor survival in a cohort of 167 ESCC patients [15]. Liu et al. showed that SAA1 protein was significantly upregulated in ESCC tissues by immunohistochemistry [16]. We further studied the function of SAA1 in ESCC cells. The loss- and gain-of-function experiments indicated that SAA1 promoted the proliferation and migration, and inhibited apoptosis of ESCC cells in vitro, consistent with previous reports in other cancers.

Next, we explored the downstream pathways of SAA1. β-catenin is the key nuclear effector of canonical Wnt signaling. The accumulation and nuclear transfer of β-catenin plays an important role in cancer progression by activating the transcription of Wnt/β-catenin target genes [33, 34]. Its phosphorylation level is closely related to the proliferation, migration and apoptosis of various tumor cells, and the role of phosphorylation at different sites in tumor progression varies. Previous studies have revealed that Ser675 is heavily phosphorylated in colon cancer cells and is associated with colon cancer progression and metastasis [35, 36]. A previous study on ESCC has shown that Aurora A promotes the invasion and metastasis of ESCC cells by improving the stability and transcriptional activity of β-catenin phosphorylated proteins at the Ser675 site [37]. In the present study, our gain- and loss-of-function results indicated that SAA1 plays an important role in regulating the phosphorylation level of β-catenin at the Ser675 site. pSer675-β-catenin was associated with SAA1-driven proliferation and migration of ESCC cells. In addition, the nuclear translocation of β-catenin and pSer675-β-catenin was significantly reduced in SAA1-knockdown Eca109 cells. However, in TE-1 cells, β-catenin and pSer675-β-catenin were localized mainly on the cell membrane, with a small amount translocated to the cytoplasm and nucleus in SAA1-overexpressing TE-1 cells. The localization of β-catenin and pSer675-β-catenin in different cell lines of the same cancer may vary. In breast cancer cell lines, β-catenin is mainly localized on the cell membrane of MCF-7 cells, while in SKBR3 cells, it is mainly localized in the cytoplasm and nucleus [38]. Cell membrane expression of pSer675-β-catenin was also observed in liver cancer cells and rat mammary gland epithelial cells [39, 40]. We are also concerned about the result of a study showing that the expression of downstream target genes of β-catenin is not completely dependent on nuclear β-catenin [36]. pSer675-β-catenin on the cell membrane may upregulate the expression of target genes in some unknown way. Moreover, SAA enhances glycerol release and lipase activity in porcine adipocytes by activating protein kinase A (PKA) [41]. β-catenin can be phosphorylated by PKA at Ser675 in human embryonic kidney cells [42]. Whether SAA1 mediates the phosphorylation of Ser675 β-catenin by activating PKA in ESCC cell lines needs to be further explored. The present study did not exclude the possibility that other phosphorylation sites of β-catenin may be involved in this regulation.

We also explored the upstream regulators of SAA1. S1P/S1PR1 signaling plays an important role in the growth, infiltration, metastasis, angiogenesis, and autophagy of various types of tumor cells by regulating the STAT, ERK, Akt and Rac pathways [21]. High expression of S1PR1 has been found to be involved in the proliferation and survival of ESCC cells by activating the STAT3 signaling pathway [43]. Our study revealed that S1P upregulated the expression of SAA1 and pSer675-β-catenin in ESCC cells, and this effect was partially mediated by S1PR1. However, whether other S1PRs are involved in this regulatory process needs to be further clarified.

## Conclusions

In summary, this study indicates that SAA1 promotes the progression of ESCC by upregulating β-catenin phosphorylation at Ser675 and that the S1P/S1PR1 pathway plays an important role in its upstream regulation. These results provide new insights into the occurrence and progression of ESCC and indicate that the S1P/S1PR1/SAA1/β-catenin axis may serve as a potential therapeutic target in ESCC.

## Data Availability

All data can be obtained from the corresponding author upon reasonable request.
